# Countering Sweet Deception: A Field Experiment Testing the Impact of Added Sugar Warning Labels on Parental Perceptions and Purchasing Preferences for Commercial Infant and Toddler Foods

**DOI:** 10.3390/nu18213673

**Published:** 2026-11-05

**Authors:** Helen G. Dixon, Maree Scully, Mamaru A. Awoke, Andrea Schmidtke, Jennifer McCann, Ashleigh Haynes, Mihiri Silva, Jane Martin, Alison McAleese, Bridget Kelly

**Affiliations:** 1 Centre for Behavioural Research in Cancer, Cancer Council Victoria, East Melbourne, VIC 3002, Australiamamaruhu@gmail.com (M.A.A.); ashleigh.haynes@cancervic.org.au (A.H.); 2 Melbourne School of Psychological Sciences, Faculty of Medicine, Dentistry and Health Sciences, The University of Melbourne, Parkville, VIC 3052, Australia; 3 Food for Health Alliance, East Melbourne, VIC 3002, Australia; andrea.schmidtke@foodforhealthalliance.org.au (A.S.); jane.martin@cancervic.org.au (J.M.); 4 Institute for Physical Activity and Nutrition (IPAN), School of Exercise and Nutrition Sciences, Deakin University, Burwood, VIC 3125, Australia; j.mccann@deakin.edu.au; 5 Inflammatory Origins, Murdoch Children’s Research Institute, Parkville, VIC 3052, Australia; mihiri.silva@mcri.edu.au; 6 Melbourne Dental School, Faculty of Medicine, Dentistry and Health Sciences, The University of Melbourne, Parkville, VIC 3052, Australia; 7 The Royal Children’s Hospital Melbourne, Parkville, VIC 3052, Australia; 8 Alison McAleese Consulting, Melbourne, VIC 3000, Australia; 9 Early Start, School of Medical, Indigenous and Health Sciences, Faculty of Science, Medicine and Health, University of Wollongong, Wollongong, NSW 2500, Australia; bkelly@uow.edu.au

**Keywords:** commercial infant and toddler foods, added sugar, warning labels, parents, natural experiment

## Abstract

Background/Objectives: Commercially produced infant and toddler foods (CITFs) often contain high levels of added sugars, which can contribute to tooth decay, a preference for sweet foods, and long-term health risks. Warning labels (WLs) signposting added sugar may help parents to evaluate CITFs and choose healthier options for their children. This study examined the effects of added sugar WLs on parents’ selection and perception of CITFs. Methods: In a between-subjects field experiment, 533 Australian parents were allocated to either an infant or toddler food arm based on their youngest child’s age, then randomly assigned to a control (no WL) or intervention (WL) condition. Participants viewed three sets of four CITFs (two with added sugars and two without) and selected the product they would be most likely to purchase for their child. Outcomes included product choices, purchase intentions, perceived added and total sugar content, and perceived healthiness. Results: Parents exposed to WLs were significantly less likely to choose CITFs with added sugar than those in the control condition (27% vs. 43%, *p* < 0.001). WLs also reduced purchase intentions (M = 2.83 vs. M = 3.28, *p* = 0.011) and perceived healthiness (M = 2.90 vs. 3.29; *p* = 0.001) of CITFs with added sugar, increased accurate identification of CITFs containing added sugar (89% vs. 61%; *p* < 0.001) and increased perceived total sugar content (M = 4.88 vs. M = 4.30, *p* < 0.001). Conclusions: In this experimental study, added sugar WLs improved parents’ understanding of added sugar content and reduced selection of, and purchase intentions for, CITFs with added sugar, suggesting they may be an effective strategy to support healthier food choices for young children.

## 1. Introduction

Nutrition during infancy and early childhood (defined here as up to age 3) is fundamental to healthy growth, cognitive development, immune function, and the prevention of chronic diseases later in life [1,2,3]. Despite the importance of early nutrition, many young children’s diets fall short of national and international guidelines. In Australia, where this study was conducted, 80% of children aged two–three years do not meet fruit and vegetable intake recommendations [4], and discretionary foods (which are high in saturated fat, added sugars and/or salt) contribute over a quarter (27%) of total energy intake among children aged 2–4 years [5].

In relation to early childhood diets, sugars, especially “free sugars” (sugars added during manufacture or preparation, plus sugars that are naturally present in honey, syrups, extruded fruit and vegetable products, including fruit juices and fruit juice concentrates [6]) are a key area of concern. Consumption of foods high in free sugars can displace more nutritious foods, reducing overall diet quality [7,8], and may establish a persistent preference for sweet foods [9,10]. In early childhood, frequent consumption of these foods is also associated with adverse health outcomes, including dental caries [11], and an increased risk of unhealthy weight gain and related chronic diseases across the life course [12]. Australian research has found that by two years of age, almost one in four children exceed the World Health Organization (WHO) recommendation that free sugars contribute less than 5% of total daily energy intake [13].

The modern food environment poses a major challenge to healthy eating in early life, with the widespread availability and aggressive marketing of nutritionally poor commercial infant and toddler foods (CITFs) [14,15]. In response to this, the WHO has recommended stronger policies to protect infants and young children from the inappropriate promotion of commercial food products and has developed nutrient and promotion profile models to support these efforts [16]. A cross-sectional audit of 330 CITFs sold in Australia found that only 28% met the compositional requirements developed by the WHO [16], with many exceeding sugar and energy thresholds and nearly a third containing added sugars [17]. All products displayed multiple claims on the packaging which are not permitted under WHO’s promotional requirements for CITFs.

Parents often perceive CITFs as healthy options and can be influenced by front-of-pack (FOP) claims such as “contains real fruit” or “no added sugar” [7,18,19]. However, these products often contain high levels of free sugars derived from processed fruit ingredients, including purees, pastes, dried powders, juices, and concentrates [20,21]. Australian research has shown that CITFs are the leading source of free sugars among children aged 12–14 months, contributing more than one-quarter (26.6%) of total free sugar intake [22]. Concerningly, a UK survey found most parents (60%) believed that natural sugars in CITF are not harmful to infants [23]. Similarly, an Australian parent poll found that many parents are strongly influenced by claims such as “natural sweetness” or “no added sugar” when choosing CITFs, suggesting they may not recognize that these products can still contain high levels of harmful sugars, including those from concentrated fruit ingredients [24]. Together, these findings highlight the need for compositional limits on sugars in CITFs to drive reformulation, complemented by clearer labelling strategies that help consumers to identify products containing added sugars and make healthier choices for their children.

Regulations governing the labelling of packaged foods, including CITFs, vary across jurisdictions. Australia currently regulates mandatory nutrition information panels and ingredients lists and sets rules for certain nutrition, health and ingredients claims but does not have a mandatory FOP nutrition labelling scheme [25,26]. The WHO recommends FOP nutritional labelling as a cost-effective “best-buy” policy tool to help consumers quickly understand the nutritional quality of food, make healthier purchasing choices, and encourage food manufacturers to reformulate products to be lower in nutrients of concern [27]. A growing number of countries have already implemented, or are considering mandating, interpretive front-of pack labelling systems, including warning labels (WLs) signposting products high in nutrients of concern [28,29]. Displaying WLs on CITFs high in added sugars offers a potential regulatory solution to help counter prolific industry-driven marketing of CITFs, and to inform and empower parents to accurately evaluate the sugar content in these products and their suitability for young children.

Systematic reviews and experimental studies show that WLs signposting sugar content, especially those combining text and graphics, effectively improve understanding, influence attitudes, and reduce the selection and purchase of sugary foods and drinks across age groups [30,31]. Prior online experimental research [23,32,33] found that WLs help parents more accurately evaluate CITFs with added sugars. One online experiment involving over 1300 Australian parents tested several different added-sugar WL designs and found that WLs, particularly those combining text with graphic icons, improved identification and perceptions of added and total sugar in CITFs and reduced intentions to purchase high-sugar products, although no significant effects of WLs on product preferences were evident [32].

However, a subsequent experiment using online choice scenarios more reflective of online shopping, and involving over 1000 Australian parents, found that displaying a WL with text plus a graphic depicting a teaspoon of sugar reduced the likelihood of choosing high-sugar CITFs (from 53% to 31%) [33]. This WL also reduced purchase intentions, and decreased perceptions of suitability, naturalness, and healthiness, while increasing perceived levels of added and total sugar. Similarly, an experiment involving over 1000 UK parents found that CITF desserts displaying sugar WLs were selected by 27.1% fewer participants than those without WLs (*p* < 0.001), and this effect was consistent across income, occupation, and education levels. The latter mixed-methods study also found via parallel focus groups that UK parents strongly supported the introduction of sugar WLs on CITFs [23], with similar views expressed by Australian parents in our qualitative research [34].

To date, most of the evidence assessing the impacts of sugar WLs on purchase-related comes from online experiments and hypothetical choice scenarios. While informative, these approaches may not fully capture the dynamic, multi-sensory nature of making real-world food choices, raising concerns about their ecological validity [35]. To strengthen the external validity of previous work, it is essential to test WLs in more naturalistic settings, which enable assessment of actual food choice behaviour, accounting for environmental cues, time pressures, and other distractions.

Accordingly, this study sought to extend prior experimental research conducted in virtual settings by observing parents’ product choices in response to a sugar WL intervention in a controlled yet realistic environment, where participants could physically interact with actual CITF products and packaging (e.g., pick them up; handle them; and view the front, back and sides of the pack if they wished). The primary aim was to test whether displaying added sugar WLs on CITFs containing added sugars reduces parents’ choice of such products, which was assessed by asking which product they would be most likely to buy from a choice set. The secondary aims were to investigate the impact of WLs on parents’ perceptions of sugar content and the healthfulness of CITFs that are high in added sugars, and their purchasing intentions (e.g., likelihood of future purchase) towards such products. We hypothesized that compared to parents who are not exposed to WLs, parents exposed to WLs on CITFs containing added sugars will be:

H1. Less likely to choose CITFs with added sugar;
H2. Better able to identify CITFs with added sugars;
H3. Perceive CITFs containing added sugar as higher in total sugar;
H4. Perceive CITFs containing added sugar as less healthy;
H5. Show reduced purchasing intentions towards such products.

## 2. Materials and Methods

### 2.1. Participants

Participants were the parent, stepparent, grandparent, foster parent or guardian (hereafter referred to as parents) of infants (6 to <12 months) and/or toddlers (12 to <36 months). Parents were excluded if they did not regularly care for the child for at least one day per week; were aged younger than 18 years; or if they or their close friends or family worked in food marketing or as a dietitian or nutritionist. Eligibility was not restricted based on the child’s consumption of commercial infant and toddler foods, breastfeeding status, or complementary feeding status. Where parents had more than one child in the eligible age range (6 to <36 months), they were instructed to answer the survey in relation to the youngest of those children. Power calculations were based on an RCT conducted by CI Smith-Taillie that tested the impact of pictorial warnings on purchases of sugary drinks for children based on parents’ purchasing behaviour in a store laboratory [36]. The target sample size was *n* = 500 participants (*n* = 125 per condition for parents of infants and parents of toddlers, respectively) which was intended to provide sufficient power to detect a difference in the magnitude observed in that study (control label: 45%; pictorial WL: 28%), with a power of 0.80 (*p* < 0.05).

### 2.2. Design and Procedure

#### 2.2.1. Recruitment

Following approval from the Cancer Council Victoria’s Human Research Ethics Committee (reference number HREC/2226ii/CCV-2024), a between-subjects field experiment was conducted between November 2024 and January 2025 at two children’s science museums in Australia (i.e., Scienceworks in Melbourne, Victoria, and the Early Start Discovery Space in Wollongong, NSW). In each setting, signs were displayed saying, “Parents and carers, seeking your input! Research study: baby and toddler foods.” and a fieldwork station was set up with a table and chairs where the study materials could be stored and participants could sit to undertake the survey should they wish. In the play space surrounding the fieldwork station, trained fieldworkers approached adults who appeared to be parents of children in the target age range and invited them to participate in “a study about baby and toddler foods”. Where parents/carers showed interest in the study, the field worker then showed them the explanatory statement and informed consent form on the i-Pad, allowing the parent to read through this themselves, or reading it out to the parent if the parent preferred. Details of the experimental manipulation and study sponsor were not disclosed to parents prior to participation to avoid priming or biasing their responses.

Parents who provided informed consent to participate were asked some initial questions to establish their study eligibility (i.e., whether they were aged 18 years or over and were the parent of child aged 6 to <36 months) and were then allocated to either the infant or toddler food study arm based on the age of their youngest child. Within each study arm, participants were randomly assigned to a WL condition: absent (control, status quo) vs. present (intervention). Group allocation was performed using the randomisation function in Qualtrics XM (version accessed between November 2024 and January 2025), the online survey software used for data collection.

#### 2.2.2. Product Stimuli

**Figure 1:**
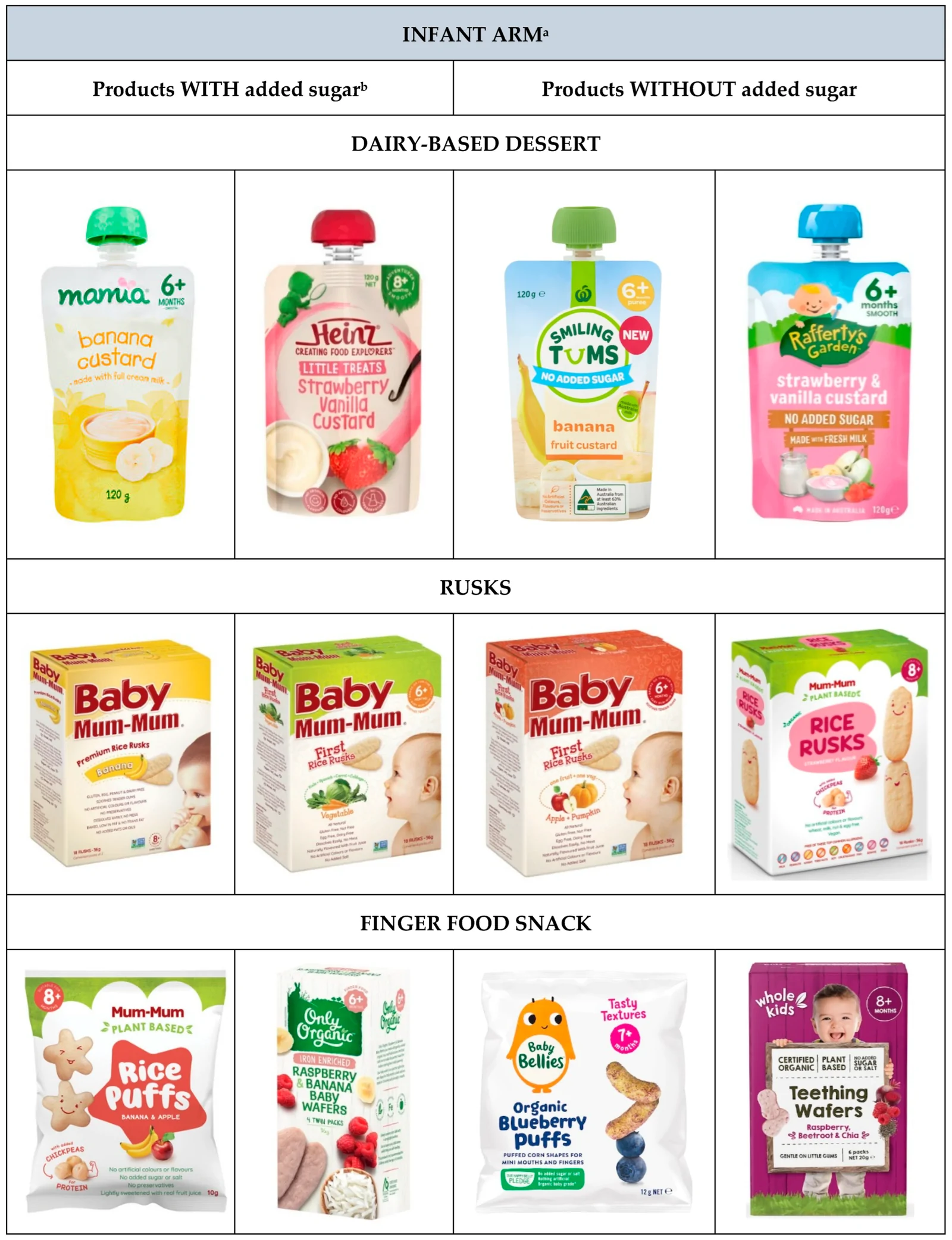

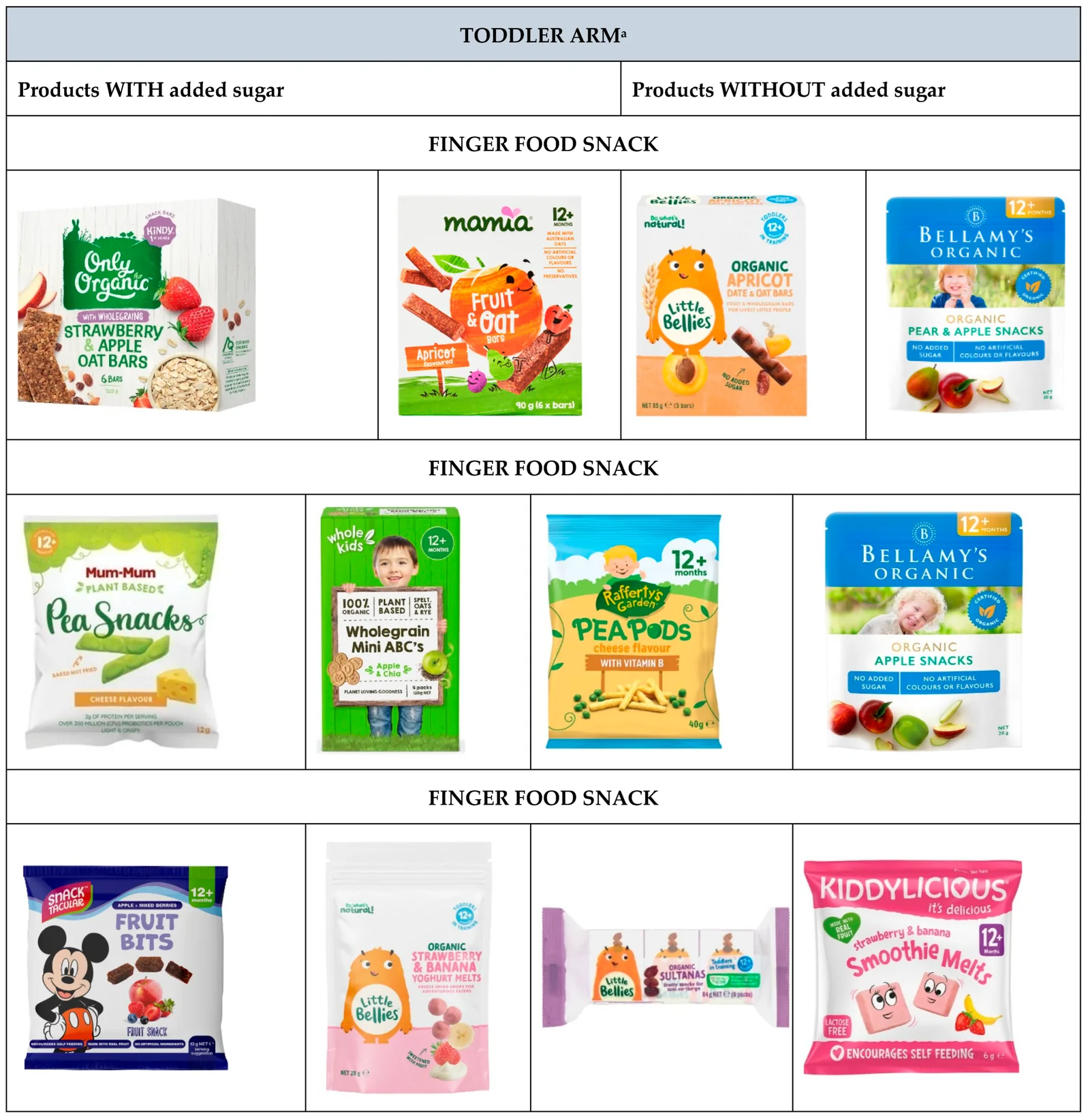
CITF product sets tested in the infant and toddler study arms (with WL intervention applied to products with added sugar). Notes: Images depict commercially available infant and toddler food products that were used as study stimuli. Images were obtained from publicly accessible Australian retailer and manufacturer websites and are included solely to illustrate the materials presented to participants and facilitate replication of the study. ^a^ Participants were assigned to the infant or toddler study arm based on the age of their youngest child. ^b^ In the intervention condition, WL stickers were applied to the top-right corner of products with added sugar. In the control condition, none of the products displayed WL stickers.

Each participant was presented with three separate sets of actual CITF products reflecting common product categories on the market in Australia. For the infant study arm, the three product sets featured were dairy-based desserts, rusks and finger food snacks, respectively. For the toddler study arm, all three sets featured finger food snacks, given that they are the dominant category on the market for this age group [17]. Every product set comprised four real CITF products (purchased for the study), two containing added sugar and two with no added sugar (see Figure 1). Product stimuli were operationalised by one of the researchers (MS) reviewing an existing database of Australian CITFs and the online websites of major Australian retailers of CITF products to identify exemplars of CITFs reflecting the featured product categories, the presence or absence of added sugars, and that were commercially available in Australia at the time of the study’s development. For the purposes of this study, added sugar was identified using the ingredient-based component of the Food Standards Code definition for use of a “no added sugar” claim. Products were classified as containing added sugar if they included ingredients such as sugar, brown sugar, caramelized sugar, maltodextrin, dextrose, glucose, golden syrup and concentrated fruit juice [37].

#### 2.2.3. Warning Label (WL) Intervention

**Figure 2:**
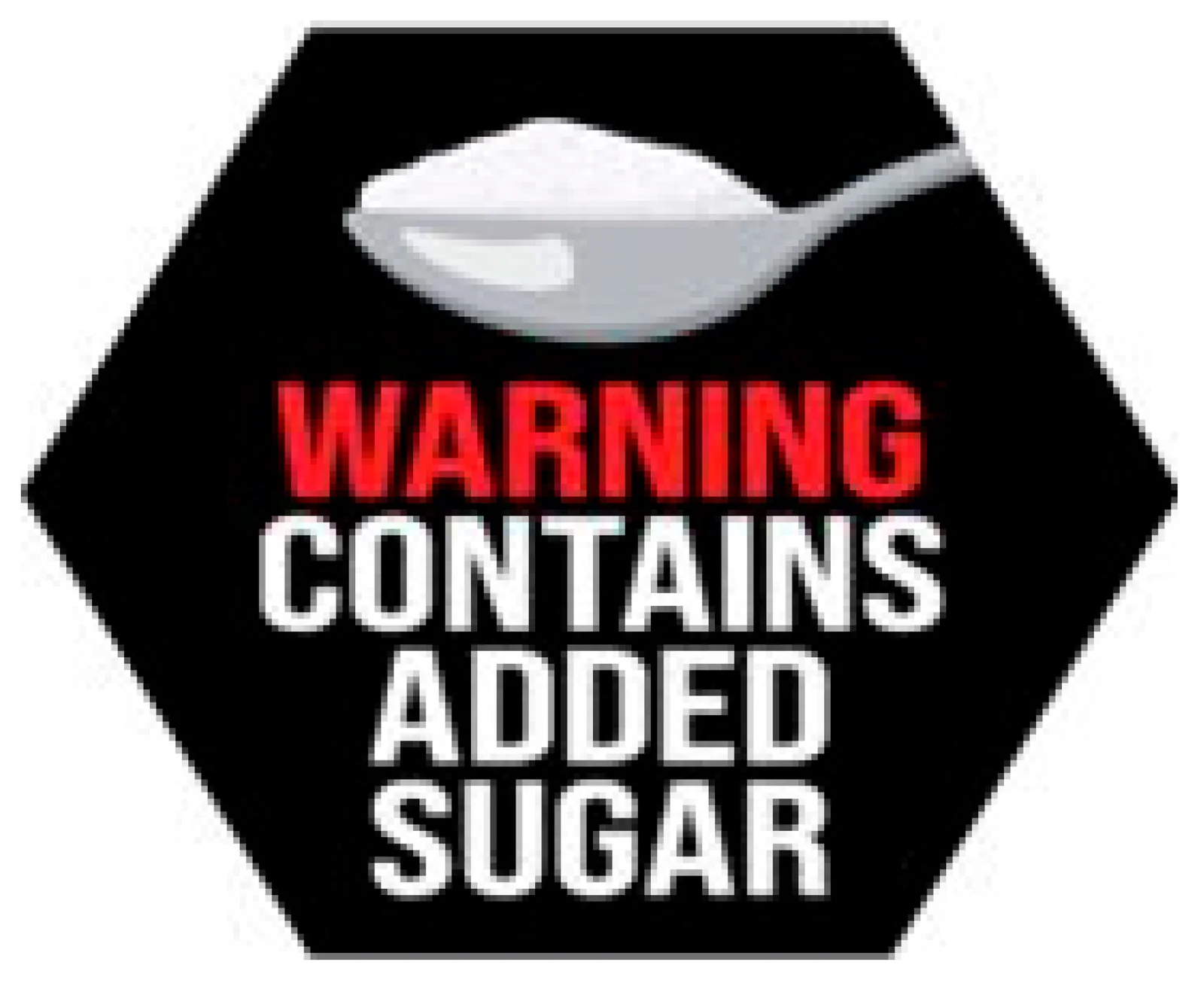
In the intervention condition, added sugar WL stickers were displayed on all CITF products containing added sugars.

Prior to viewing product stimuli, participants in the intervention condition were shown a showcard displaying the WL design accompanied by the following brief explanation: “This product contains added sugar. Baby and toddler foods containing added sugar should be limited or avoided.” This was intended to mimic supporting communications (esp. point-of-sale signage) that would typically accompany the introduction of a new FOP labelling scheme in Australia. For the WL intervention group, all product stimuli that contained added sugars displayed a WL sticker on the top corner of the FOP (see Figure 2).

#### 2.2.4. Product Choice Task

For the product choice task, product sets were presented to participants in a random order (both between and within sets) on a small tray with WLs displayed according to condition. Although not instructed to do so, participants were able to pick up the products to look at other sides of the packaging to inform their selections. This enabled them to view the ingredient list and nutrition information panel (NIP), which both appear on the back or side of product packaging in Australia, as well as the front of the pack should they wish. Participants were instructed to assume that all products were of equivalent serving size and cost when completing the product choice tasks.

#### 2.2.5. Product Perception Ratings

After completing the product choice task, participants were randomly assigned one of the six CITFs with added sugar from their study arm, which they were asked to view and rate on a series of response measures. Participants’ responses were recorded by fieldworkers on a tablet device using an online survey programmed in Qualtrics.

#### 2.2.6. Debrief

At the conclusion of the survey, participants were debriefed on the study aims and sponsor. They also received a healthy snack for their child (fresh banana) along with a brochure containing summary information on healthy eating for infants and toddlers, based on the Australian Dietary Guidelines for Infants/Children [38] and the Raising Children Network [39] guidelines.

### 2.3. Measures and Outcome Variables

#### 2.3.1. Socio-Demographics

Participants’ gender, age, highest level of educational attainment, parental status (sole or co-parent), parentship (parent, grandparent/guardian), whether a language other than English was spoken at home (yes or no), and the number of children in the household were recorded.

#### 2.3.2. Usual Consumption of Commercial Infant and Toddler Foods (CITFs)

Parents were asked, “Thinking about a typical week, how often does this child eat ready-made baby or toddler foods? By ready-made baby or toddler foods, we mean baby or toddler foods that you can buy pre-made. These foods usually come packaged in pouches, jars or boxes. For example, purees, ready-made meals and handheld snacks. They do not include baby or toddler formula.” The response options were as follows: Every day of the week; 4 to 6 days a week; 2 to 3 days a week; one day a week; less than one day a week; never.

#### 2.3.3. Perceived Importance of Factors Influencing Food Choices for Children

Participants were asked to indicate how important each of the following factors are when choosing food for their child: quick and easy; healthy; child likes the taste; price; child likes the packaging; non-perishable; trusted brand. Response options included “not at all important”, “somewhat important”, “moderately important”, “very important” and “extremely important”.

#### 2.3.4. Product Choice Measure

To assess WLs’ impacts on purchasing decisions, participants were presented with three separate sets of four CITFs (two with no added sugar and two with added sugar) that reflected their assigned WL condition and asked, “If you were choosing food for your [baby/toddler], which of these products would you be most likely to buy?”. Responses to each task were coded as a binary outcome indicating whether the participant selected one of the two CITFs with added sugar in the set.

#### 2.3.5. Product Perceptions Measures

Next, participants were randomly assigned one CITF product from their study arm (infant/toddler food) that contained added sugar and asked to rate it based on the following outcomes.

Added sugar

Participants were asked whether they thought the product contained added sugar. Responses of “yes” were coded as correct, while “no” and “not sure” were coded as incorrect.

Total sugar

Using a 7-point scale, participants rated their perception of how much total sugar the product contained (1 = “no sugar” to 7 = “high sugar”).

Healthiness

Using a 7-point scale, participants rated their perception of how healthy the product was (1 = “not healthy at all” to 7 = “very healthy”).

Purchase intentions

Participants were asked to rate their likelihood of purchasing the CITF product with added sugar for their child if it were available in the supermarket where they normally shop. Responses were recorded on a 7-point scale ranging from 1 = “very unlikely” to 7 = “very likely”.

### 2.4. Statistical Analysis

Data were analyzed using Stata/MP V.16.1 (StataCorp). To assess the effect of the WL intervention on product choice, a mixed-effects logistic regression model was conducted, with the intercept specified as a random effect at the participant level to account for repeated measures across product categories. A binary logistic regression model was used to analyze the outcome assessing correct identification of CITF products containing added sugar. Continuous outcomes (purchase intentions, perceived total sugar content and perceived healthiness) were analyzed using linear regression models. All models adjusted for study arm (infant/toddler) and data collection site (Scienceworks/Early Start Discovery Space). Marginal predicted probabilities (for binary outcomes) and marginal means (for continuous outcomes) from these models are reported.

For each outcome, exploratory analyses were conducted to test for interactions between WL condition (control/intervention) and selected socio-demographic characteristics (education, parental status, language other than English spoken at home, frequency of infant/toddler’s CITF consumption, and number of children in the household) that have previously been associated with CITF purchase preferences and intentions [19,24]. However, as no significant interactions (*p* < 0.05) were observed, these analyses are not reported.

## 3. Results

### 3.1. Sample Characteristics

#### 3.1.1. Demographics

A total of 533 parents participated, with the socio-demographic profile of the sample generally balanced across WL condition (see Table 1). The sample was predominantly female (86%), with the majority aged 30 to 39 years (71%). A substantial proportion (81%) had attained tertiary-level education. Most participants (85%) were co-parents, while around one in ten (11%) were a grandparent or guardian. A majority (73%) spoke only English at home.

#### 3.1.2. Frequency of CITF Consumption

Half of the participants (50%) reported that their infant/toddler consumed CITFs on four or more days per week (see Table 1).

#### 3.1.3. Perceived Importance of Various Factors When Choosing Food Products for Their Infant/Toddler

Most participants (91%) rated healthiness as extremely/very important when choosing products for their infant/toddler, followed by whether their child liked the taste (76%), highlighting that healthiness is the primary consideration in parental food choices. Only about one-third of parents considered convenience (37%), being a trusted brand (36%) and price (32%) as extremely/very important, although most still considered these factors somewhat/moderately important (60%, 47% and 58%, respectively). Practical aspects such as non-perishability were of moderate concern (17% extremely/very important; 59% somewhat/moderately important), suggesting they influence decisions to a lesser degree. Notably, whether their infant/toddler liked the packaging was generally considered not as important (82% not at all important). Overall, these findings demonstrate that parents’ purchasing preferences for CITFs are strongly health-oriented, with taste, branding and affordability playing secondary roles, and minimal influence from infant/toddler product packaging preference.

### 3.2. Effects of WL Intervention

#### 3.2.1. Product Choice (H1)

**Figure 3:**
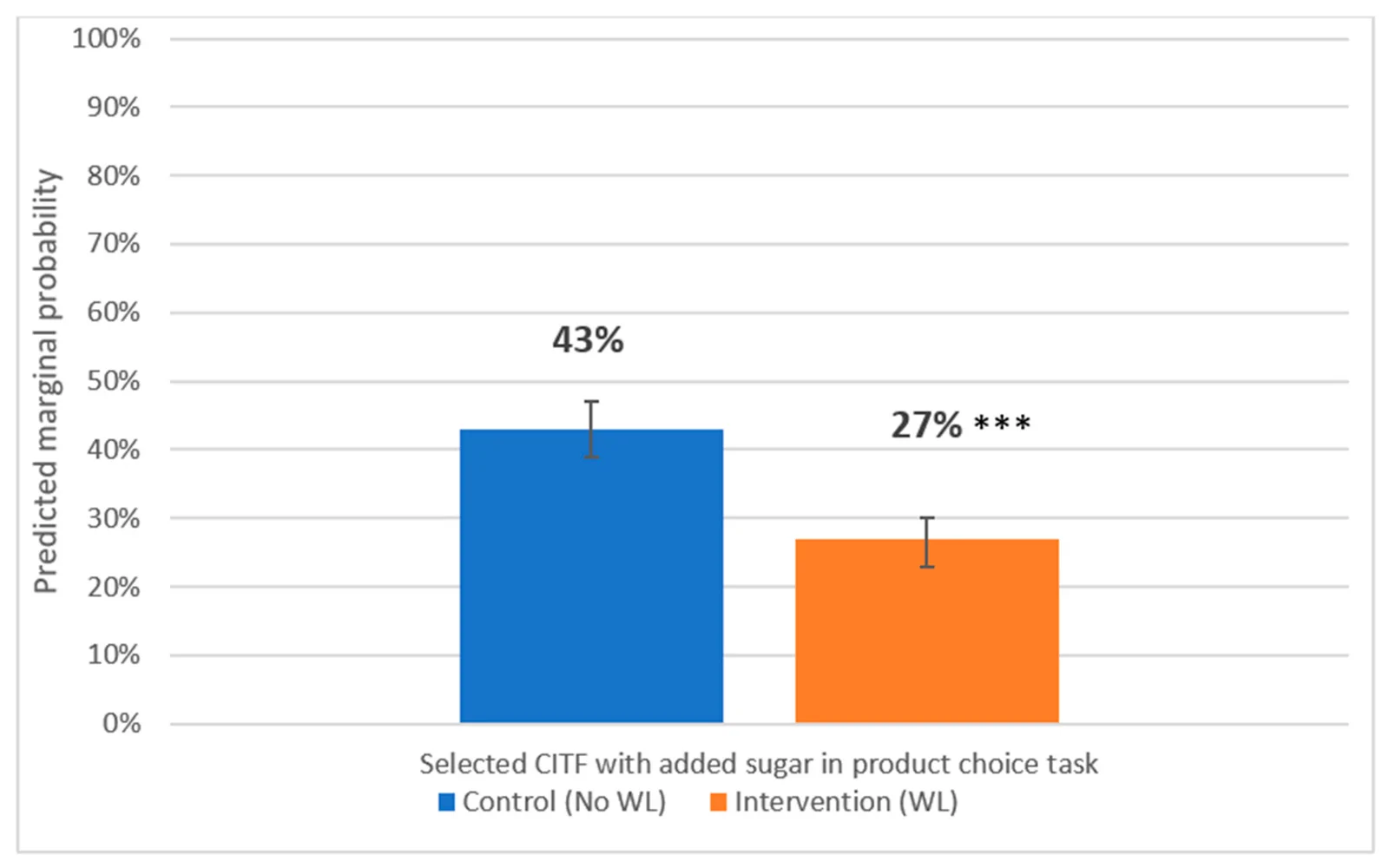
Marginal predicted probability of selecting CITF product with added sugar in the product choice task by WL condition (*n* = 533). Notes: *** *p* < 0.001. The error bars indicate the 95% confidence interval of the marginal predicted probability.

Across the three choice tasks, participants had significantly lower odds of selecting a CITF product with added sugar when WLs were displayed compared to the control condition (OR = 0.43, 95% CI: 0.33–0.57, *p* < 0.001). Specifically, the probability of choosing a product with added sugar dropped from 43% in the control group to 27% in the intervention group (Figure 3). (See Supplementary Table S1 for detailed results of the mixed-effects logistic regression model.)

#### 3.2.2. Product Perceptions

The following product perceptions were assessed in response to a single randomly assigned CITF product that contained added sugar, with the WL displayed for participants in the intervention condition only.

Identification of Products Containing Added Sugar (H2)

**Figure 4:**
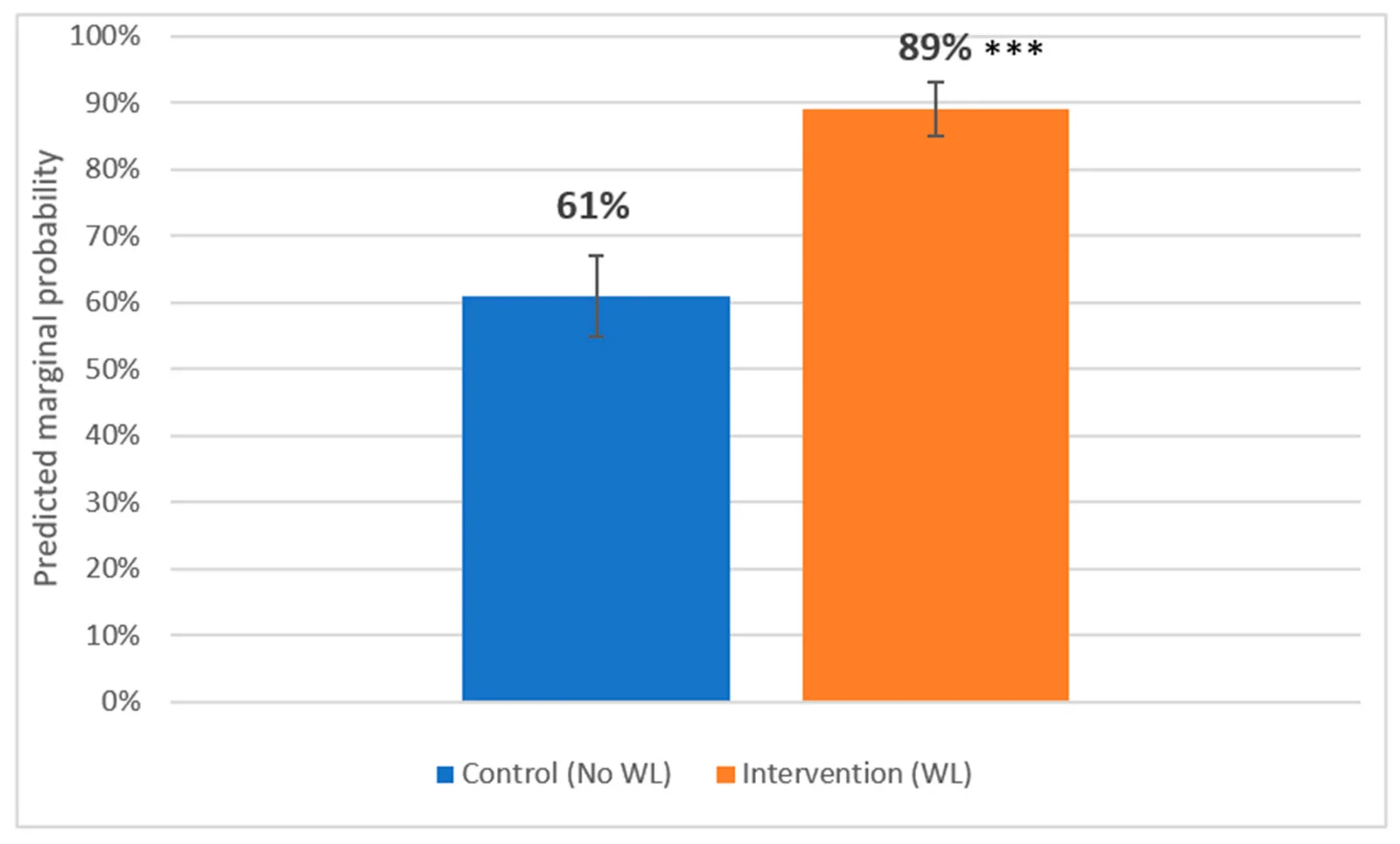
Marginal predicted probabilities of correctly identifying CITF product as containing added sugar, by WL condition (*n* = 533). Notes: *** *p* < 0.001. The error bars indicate the 95% confidence interval of the marginal predicted probability.

When shown a single CITF that contained added sugar, the odds of participants correctly identifying that the product contained added sugar were five times as high among participants in the WL condition compared to the control (no WL) condition (89% vs. 61%; OR = 5.05, 95% CI: 3.21–7.95, *p* < 0.001; Figure 4). (See Supplementary Table S2 for detailed results of the binary logistic regression model.)

Total Sugar (H3)

**Figure 5:**
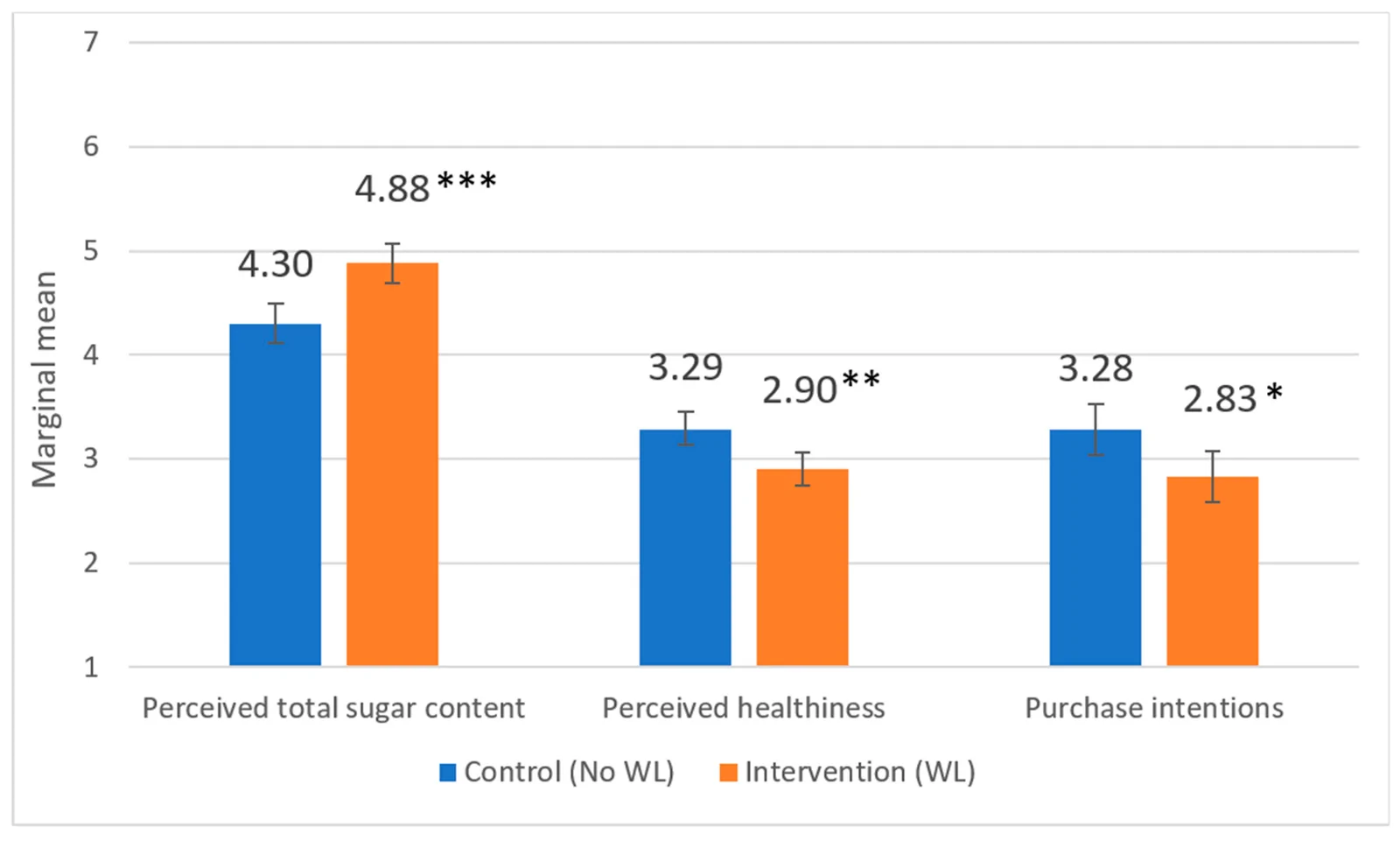
Marginal predicted mean scores (1–7 scale) of participants’ ratings of CITFs with added sugar on perceived total sugar content, perceived healthiness and purchase intentions, by WL condition (*n* = 533). Notes: *** *p* < 0.001, ** *p* < 0.01, * *p*< 0.05. Error bars indicate a 95% confidence interval of the marginal mean.

As Figure 5 illustrates, when CITFs with added sugar displayed a WL, participants perceived them as having significantly higher total sugar content than those without a WL (M = 4.88 vs. M = 4.30; β = 0.58, 95% CI: 0.31–0.85; *p* < 0.001).

Healthiness (H4)

Participants’ perceptions of the overall healthiness of CITFs with added sugar varied by WL condition, with ratings significantly lower when a WL was shown on the FOP compared to when no WL was present (M = 2.90 vs. M = 3.29; β = –0.38, 95% CI: –0.61 to –0.16, *p* = 0.001).

Purchase Intentions (H5)

As shown in Figure 5, purchase intentions significantly differed by WL condition. Participants reported lower intentions to buy CITFs with added sugar when they displayed a WL compared to when no WL was present (M = 2.83 vs. M = 3.28; β = −0.45, 95% CI: −0.80 to −0.10; *p* = 0.011).

## 4. Discussion

Consistent with hypotheses, this field experiment yielded clear evidence that FOP WLs signposting added sugars improve parents’ ability to identify CITFs with added sugars, reduce their likelihood of choosing such products for their young children, and reduce intentions to purchase CITFs containing added sugars. WLs also increase perceptions of the level of sugar in these products and reduced parental perceptions of the overall healthiness of CITFs containing added sugars. Together, these findings demonstrate that WLs offer an effective public health strategy to inform parents about added sugars in CITFs and promote healthier food choices for their infants and toddlers. Our findings converge with accumulating evidence across other food product categories and population groups that support the use of WLs to help consumers identify and evaluate products high in nutrients of concern, ultimately promoting healthier food and drink choices [30,31].

### 4.1. WLs Reduce Preferences and Intentions Towards Sugary CITFs

We deliberately designed this field experiment to assess the effects of WLs on parent’s perceptions and preferences towards actual CITF products, thereby strengthening findings from previous online experimental simulations where WLs were applied to fictional CITF products high in added sugars [32,33]. The finding that WLs effectively steered parents away from CITFs with added sugar towards healthier options aligns with a growing body of literature showing that interpretive WLs can effectively reduce parents’ selection of unhealthy products for their children, particularly sugar-sweetened beverages [23,36,40] and snacks [40]. The observed reduction in parents’ purchase intentions for CITFs with added sugar further supports the behavioural impact of WLs, aligning with experimental studies showing that WLs can reduce purchase intentions for foods and drinks high in added sugar [40,41], especially pictorial (rather than text only) WLs [42].

### 4.2. WLs Influence Product Perceptions

Prior research has shown that parents’ product choices and perceptions can be influenced by on-pack marketing claims on CITFs, and that they seek more transparent labelling of these products [18,19,34]. In the present study, we found that parents’ perceptions of the level of both added and total sugar in CITFs high in added sugar increased significantly when they viewed WLs on the FOP of CITFs, mirroring findings from a recent mixed-methods study (online survey and focus groups) which reported that adding sugar WLs to infant food products assisted parents in identifying products high in sugar [23]. This indicates that WLs can enhance parents’ understanding of product nutrition composition and quality and support informed decision-making. Consistent with our results, previous meta-analyses and systematic reviews have shown that FOP labels, particularly those featuring interpretative text such as “high in sugar” and visual warning signs are the most effective strategies for improving consumers’ (aged over 16 years) accurate understanding of sugar content in packaged foods [30]. Moreover, these labels have been found to influence purchasing behaviour by prompting consumers to select products with lower sugar content. WLs may also help correct common misconceptions about the sugar content of CITFs, which are often perceived as healthier than they actually are due to marketing tactics and on-pack claims [43,44]. WLs act as salient cues that signal nutrient risk (i.e., “this product has added sugar”) in a way that non-interpretive nutrient information may not. This raises a perceived “red flag” about sugar, eliciting stronger judgments of unhealthfulness and likely reducing the appeal of the product. Studies testing FOP WLs on beverages show similar shifts in risk perception, emotional response, and reduced purchase intentions [23,45,46,47,48].

### 4.3. WLs Detract from Perceptions of Product Healthiness

The reduction in perceived healthiness of high-sugar CITFs among participants exposed to WLs is notable for demonstrating WLs can effectively disrupt the health-halo effect often associated with CITFs, which are frequently marketed as “organic,” “natural”, “made with fruit” [43,44] and “free from” certain harmful ingredients [49], despite containing high levels of sugar. Previous randomized controlled trials have similarly shown that pictorial health warnings reduce parents’ perceptions of sugar-sweetened beverages as healthy and lower their intentions to serve such products to their children [36]. Displaying added sugar WLs on CITFs simplifies the process of evaluating products’ healthiness, compared with reading ingredient lists and NIP that require nutrition literacy and time to interpret [50]. WLs may therefore support parents in making healthier purchasing decisions.

Although some studies have highlighted that WLs do not completely eliminate the influence of nutrient content claims [47], WLs signposting products high in nutrients of concern may prompt parents to avoid such products (in this case CITFs) even when the packaging simultaneously displays health, nutrient or other marketing claims. A recent scoping review found that parents often place trust in foods labelled as suitable for babies, and are frequently influenced by claims such as “sweetened with fruit” or “natural sugar,” which many interpret as indicating low sugar content or a healthier product, even when free sugars from ingredients such concentrated fruit juice are present [51]. The review also found that some parents viewed existing sugar labelling as misleading and expressed a desire for clearer information about sugar content. Parents in Australia and the UK show strong support for clearer sugar labelling and related regulatory reforms to help them make more informed food-purchasing decisions [23,34].

A previous online experiment [32], along with the present field experiment, found that displaying added sugar WLs (that combine text with icons) can reduce parents’ positive perceptions and purchasing intentions for high-sugar CITF products. The present study applied WLs to actual CITF products that featured common packaging designs (e.g., squeezy pouches, boxes, sachets) and on-pack claims such as “no artificial colours or flavours”, “made with real fruit” and “preservative free”. These findings demonstrate that added sugar WLs can help parents make more informed and healthier choices, even in the context of competing on-pack claims that may create a misleading “health halo” over CITFs containing added sugars.

### 4.4. Parental Considerations When Choosing Foods for Their Child

Parents are the primary gatekeepers of family food purchases [52] and, perhaps unsurprisingly, we found that parents rated healthiness as an extremely or very important factor when selecting foods for their infant or toddler. However, their children’s taste preferences were also a key parental consideration, underscoring the risk that children (who have an innate preference for sweet tastes, which may be reinforced by repeated exposure to sweet-tasting foods [10]) may reinforce parents’ ongoing purchasing of high-sugar CITFs once they have experienced sweet taste. Additionally, brand reputation, convenience attributes (quick and easy; non-perishable) and price were cited as moderately important considerations in parents’ purchasing decisions (consistent with other research on drivers of parents’ food decisions [53]). In the present field experiment, we featured popular brands of CITFs as the product stimuli, simply manipulating the presence or absence of WLs on those products that contained added sugars. Notably, even though parents entered the study with prior experience of CITF brands and their children’s taste preferences, the presence of WLs appeared to over-ride these influences, significantly reducing both preferences and intentions to purchase CITFs with added sugars for their young children.

### 4.5. WLs Effective for Signposting Added Sugars in CITFs

Collectively, these findings support the implementation of mandatory WLs on CITFs as a strategy to reduce sugar intake and promote healthier eating habits from an early age. Such labelling could serve as a corrective tool, helping parents make more informed decisions by presenting clear, interpretive information about added sugar. The WHO (2022) [16] recommends not only stricter regulation of sugar content and the removal of marketing claims on CITFs but also recommends a FOP indicator on CITFs with high total sugar, which directly supports the use of mandatory WLs. Our findings, alongside growing empirical evidence, reinforce the policy recommendation for mandatory WLs on CITFs.

### 4.6. Strength and Limitations

This study has several strengths. Firstly, ecological validity was enhanced through conducting a field experiment in settings frequented by families with young children, where participants could handle actual CITF products when appraising them and making choices. Second, the random assignment of participants to WL conditions strengthens internal validity. Third, the inclusion of a range of product categories and real CITFs with pre-existing FOP claims enabled a robust test of WLs under natural conditions. Fourth, the study assessed multiple outcomes, including product choice, purchase intention, and perceptions of sugar content and healthiness, providing a comprehensive understanding of the effects of WLs. Finally, the integration of a showcard explanation for WLs mimics real-world implementation scenarios, where educational materials often accompany new labelling policies.

Despite these strengths, several limitations should be noted. Firstly, the use of real products meant that participants could have had prior knowledge or familiarity with the brands featured that may have influenced their product perceptions and preferences. Nonetheless, effects of the WL manipulation were found, consistent with our earlier WL experiments where mock products were used to protect against prior brand or product knowledge potentially confounding the effect of the WL intervention [32,33].

Second, while the sample was recruited from two different locations and participants were broadly similar to the Australian population with respect to language spoken at home, family structure and the age of parents of young children, women and individuals with tertiary education were over-represented [54,55,56,57]. This may be an artefact of our study being conducted in two children’s science museums, one of which is located within a university. While these settings enabled us to access parents of young children to recruit as participants in our experiment, these settings did not represent places where parents usually do their grocery shopping (i.e., supermarkets).

Third, the study measured immediate responses to WLs and did not assess the effects of longer-term or repeated exposure. Finally, while participants could view NIPs and ingredient lists, their engagement with this information was neither prompted nor controlled and may have been limited due to the distraction of supervising young children during the study (although this scenario may be considered analogous to the experience of parents shopping with their young children present).

### 4.7. Policy Implications

These findings have important implications for public health policy and regulatory action, as they suggest that applying WLs to CITFs could be an effective regulatory lever to nudge parents toward healthier options (lower sugar) and improve early childhood nutrition. As health professionals are among the most trusted sources of infant-feeding advice for Australian parents [58], WLs may also serve as a practical communication tool for health professionals, providing a simple and credible way to discuss product healthfulness and support evidence-based feeding recommendations [38] with parents and caregivers. Clear sugar WLs can enhance transparency, counter misleading marketing claims, and help caregivers make more informed choices. This aligns with broader nutrition labelling policies aimed at improving child health and supports the WHO’s recommendations for CITFs. Ideally, high-sugar products would not be permitted to be marketed as suitable for infants and toddlers. However, in contexts where this does occur, WLs may represent a promising and scalable approach to helping caregivers identify less healthy products and protect young children’s dietary health.

## 5. Conclusions

Displaying FOP WLs on CITFs helped parents more accurately evaluate the presence of added and total sugar content in CITFs and influenced their perceptions of product healthfulness. Products with WLs were consistently viewed as less healthy, which reduced parents’ preference for products containing added sugar and significantly lowered their intention to purchase such products. These findings suggest that FOP sugar WLs can be an effective policy tool to support informed decision-making, reduce added sugar intake and promote healthier eating habits from an early age.

## Figures and Tables

**Table 1:** Socio-demographic characteristics of participant sample (*n* = 533).

Characteristic	Total (*n* = 533)	Control (No WL) (*n* = 267)	Intervention (WL) (*n* = 266)
	*n* (%)	*n* (%)	*n* (%)
Gender ^a^			
Man or male	74 (13.9)	36 (13.5)	38 (14.3)
Woman or female	457 (85.9)	229 (86.1)	228 (85.7)
Non-binary	1 (0.2)	1 (0.4)	0 (0)
Age			
18–29 years	37 (6.9)	16 (6.0)	21 (7.9)
30–39 years	380 (71.3)	187 (70.0)	193 (72.6)
40+ years	116 (21.8)	64 (24.0)	52 (19.5)
Highest level of education ^b^			
Did not complete tertiary	100 (18.9)	45 (17.1)	55 (20.8)
Tertiary or higher	428 (81.1)	218 (82.9)	210 (79.2)
Parental status ^c^			
Sole (single) parent	20 (3.8)	8 (3.0)	12 (4.5)
Co-parent	452 (85.0)	228 (85.7)	224 (84.2)
Grandparent/guardian	60 (11.3)	30 (11.3)	30 (11.3)
Language spoken at home ^d^			
English	388 (72.9)	188 (70.7)	200 (75.2)
Other language	144 (27.1)	78 (29.3)	66 (24.8)
Number of children in household			
One	222 (41.7)	109 (40.8)	113 (42.5)
Two	248 (46.5)	126 (47.2)	122 (45.9)
Three or more	63 (11.8)	32 (12.0)	31 (11.7)
Frequency of CITF consumption			
≤1 day/week	142 (26.6)	76 (28.5)	66 (24.8)
2 to 3 days/week	127 (23.8)	64 (24.0)	63 (23.7)
≥4 days/week	264 (49.5)	127 (47.6)	137 (51.5)
Data collection site			
Discovery Centre	225 (42.2)	112 (41.9)	113 (42.5)
Scienceworks	308 (57.8)	155 (58.1)	153 (57.5)
Study arm			
Infant	109 (20.5)	55 (20.6)	54 (20.3)
Toddler	424 (79.5)	212 (79.4)	212 (79.7)

Notes: Percentages may not sum to 100% due to rounding. ^a^ *n* = 1 did not indicate their gender; ^b^ *n* = 5 did not report their highest level of education, ^c^ *n* = 1 parent did not specify if they were a sole parent or co-parenting, ^d^ *n* = 1 did not report language spoken at home.

## Data Availability

The datasets used and/or analyzed during the current study are available from the corresponding author on reasonable request.

## Supplementary Materials

The following supporting information can be downloaded at https://www.mdpi.com/article/10.3390/nu18213673/s1, Supplementary Table S1: Mixed-effects logistic regression model results for product choice. Supplementary Table S2: Binary logistic regression model results for identification of products containing added sugar.

## Abbreviations

CITFs	Commercially produced infant and toddler foods
WHO	World Health Organization
WL	Warning label
