# Supplementary material for: Countering Sweet Deception: A Field Experiment Testing the Impact of Added Sugar Warning Labels on Parental Perceptions and Purchasing Preferences for Commercial Infant and Toddler Foods
Source: Nutrients. 2026 Nov 5;18(21):3673. doi: 10.3390/nu18213673 (PMC13765693; doi:10.3390/nu18213673)
Supplement: Supplementary file 1 [file nutrients-18-03673-s001.zip › nutrients-4619511-supplementary.pdf]

Supplementary Table S1: Mixed effects logistic regression model results for product choice.

**Variable**	**β**	**SE**	**OR**	**95% CI**	***P value***
Warning label condition (intervention vs. control)	-0.84	0.14	0.43	0.33-0.57	<0.001
Study arm (toddler vs. infant)	0.45	0.18	1.57	1.10-2.22	0.012
Data collection site (Scienceworks vs. Early Start Discovery Space)	-0.55	0.14	0.58	0.44-0.76	<0.001
Constant	-0.38	0.19	-	-	0.049
Random effects					
Participant-level random intercept variance	0.76	0.21	-	-	-

Notes: *SE, standard error; OR, odds ratio; CI, confidence interval. Model estimated using mixed-effects logistic regression with a participant-level random intercept to account for repeated measures across product categories.*

Supplementary Table S2: Binary logistic regression model results for identification of products containing added sugar.

**Variable**	**β**	**SE**	**OR**	**95% CI**	***P value***
Warning label condition (intervention vs. control)	1.62	0.23	5.05	3.21-7.95	<0.001
Study arm (toddler vs. infant)	-0.33	0.27	0.72	0.42-1.23	0.228
Data collection site (Scienceworks vs. Early Start Discovery Space)	-0.17	0.22	0.85	0.56-1.29	0.440
Constant	0.81	0.29	-	-	0.005
Model fit statistics: *Pseudo* R^2 *= 0.098; AUC = 0.703*					

Notes: *SE, standard error; OR, odds ratio; CI, confidence interval; AUC, area under the receiver operating characteristic curve (ROC).*
